# Outcome Comparison of Endovascular and Open Surgery for the Treatment of Acute Superior Mesenteric Artery Embolism: A Retrospective Study

**DOI:** 10.3389/fsurg.2022.833464

**Published:** 2022-03-14

**Authors:** Wenrui Li, Saisai Cao, Zhiwen Zhang, Renming Zhu, Xueming Chen, Bin Liu, Hai Feng

**Affiliations:** ^1^Affiliated Beijing Friendship Hospital, Capital Medical University, Beijing, China; ^2^Peking University People's Hospital, Beijing, China

**Keywords:** superior mesenteric artery embolism, endovascular treatment, opening surgery, acute mesenteric ischemia, percutaneous mechanical thrombectomy

## Abstract

**Background:**

Few centers have adopted endovascular revascularization for the treatment of superior mesenteric artery embolism (SMAE). We sought to evaluate the efficacy of endovascular therapy for the treatment of SMAE and identify post-treatment prognostic factors.

**Methods:**

The clinical data of 41 patients with acute SMA embolism between 2013 and 2021 were retrospectively reviewed. Patients with mesenteric artery thrombosis, mesenteric venous thrombosis, and who had only conservative treatment were excluded.

**Results:**

Forty-one consecutive patients were identified with SMAE (median age, [range] 35–86 years). Endovascular therapy was initiated in 14 patients with no clinical evidence of bowel necrosis, with mainly mechanical thrombectomy, and technical success was achieved in 93%. Endovascular therapy had advantages in duration surgery time, blood loss, bowel rest time, ICU time, and ventilator use. There was no difference in bowel necrosis, length of necrotic bowel resected, or in-hospital mortality between the two groups. An initial white blood cell (WBC) count >15 × 103/dl and neutrophil count >13 × 103/dl were associated with an increased risk of bowel necrosis, and an initial WBC count, renal function, American Society of Anesthesiologists (ASA >3) and necrotic bowel >2 m were associated with increased mortality.

**Conclusions:**

Endovascular treatment has altered the management of SMAE, and it may be adopted in selected patients who are not at risk for bowel necrosis. Avoidance of bowel necrosis patients and close monitoring for bowel necrosis are important.

## Background

Acute mesenteric ischemia (AMI) is one of the emergencies of vascular surgery and one of the most dangerous acute abdomens. The cause is that the mesenteric arteries or veins are blocked, leading to sudden interruption of blood supply or venous return, causing blood supply disorders and malnutrition in the intestinal tract, and eventually, intestinal function loss and infarction will occur. AMI has an insidious onset, rapid progression, and serious consequences. If it cannot be diagnosed and treated on time, the mortality rate can reach 50–70% (1). Superior mesenteric artery embolism (SMAE) is the most common cause of AMI and usually occurs as an end result of cardiac arrhythmia (e.g., atrial fibrillation), left atrial thrombosis, aortic calcification, previous stenotic lesions, and tumors (2). With the improvement of modern diagnosis and treatment technology, new breakthroughs and advancements have been brought to the treatment of SMAE, such as percutaneous mechanical thrombectomy, which has been reported for the treatment of SMAE in the form of case reports and case series; these initial studies demonstrate feasibility; however, whether endovascular therapy should be the primary treatment for SMAE is still controversial (3, 4).

The goals of this study were to analyze the management outcomes of SMAE at the authors' institution, to evaluate the effect of endovascular therapy, and to identify prognostic factors associated with mortality in the treatment of SMAE.

## Methods

This study was an institutional review board-approved study evaluating current treatment for SMAE. A single-institutional procedural database was queried for all consecutive cases of SMAE treated with surgery from March 2013 to August 2021. Patients with embolic etiology for AMI were included. Patients presenting with AMI secondary to the following conditions were excluded: mesenteric artery thrombosis, mesenteric venous thrombosis, non-occlusive mesenteric ischemia, aortic dissections complicated by visceral ischemia, and visceral ischemia occurring as part of an investigational device exemption protocol. Patients who had only conservative treatment were excluded. The diagnosis of etiology (thrombotic or embolic) was based on surgeon interpretation of the clinical presentation, radiographic findings, and operative findings.

Previous diagnoses were used to establish conditions, such as hypertension, diabetes, hyperlipidemia, smoking history, chronic obstructive pulmonary disease, chronic renal insufficiency, coronary artery disease, congestive heart failure, atrial fibrillation, and cardiac valvular disease. Symptoms on presentation, preoperative imaging, laboratory values, and American Society of Anesthesiologists (ASA) class on admission were recorded. CT was recorded as positive if it confirmed arterial occlusion.

All of these patients underwent emergency surgery, and the treatment administered was categorized as “endovascular first” or “open surgery” therapy. The endovascular algorithm included attempts at endoluminal revascularization with or without the need for laparotomy. Open surgery therapy included laparotomy with an assessment of mesenteric vasculature, surgical embolectomy, bypass graft, and bowel assessment for viability. Initial endovascular treatment was indicated selectively in patients without evidence of bowel gangrene (e.g., rebound tenderness on physical examination; free air, pneumatosis intestinalis, or mesenteric venous air on CT scan). For patients who had signs of bowel gangrene, open surgery was performed. The time from symptom onset to treatment was based on the patient's duration of pain and included the diagnostic evaluation before revascularization.

## Endovascular Treatment

Endovascular therapy included mechanical thrombectomy, balloon dilatation, thrombolysis, and stent implantation. Femoral and brachial access were both used for endovascular therapy. Once the superior mesenteric artery (SMA) was confirmed, mechanical thrombectomy was used to achieve initial reperfusion of the viscera. Mechanical thrombectomy was performed with the 6F Rotarex System (Straub Medical, Wangs, Switzerland), and small careful forward and backward passages were slowly performed once or twice. Thrombolysis was initiated based on residual arterial occlusions. Thrombolysis was performed with a multiside hole infusion catheter; the catheter was positioned across the SMA origin beyond the first jejunal branches. Heparin was administered uniformly through the arterial sheath. Vasodilator adjuncts and papaverine were also recorded.

After the initiation of thrombolytic therapy, the decision to continue thrombolysis was determined by the patient's overall condition and the initial response. Therapy was continued for 1 day in patients with residual arterial occlusions; in these cases, therapy was directed at occluded vessels by positioning the catheter or infusion wire into specific side branches (ileum, ileocolic, or right colic arteries).

## Open Surgery

The decision to perform a laparotomy was determined by the patient's clinical status, physical examination, and laboratory values, but ultimately, it was the decision of the operating staff vascular surgeon and colorectal surgeon. The staff colorectal surgeon also made the decision of bowel viability and the length of the bowel to resect. The omentum and transverse colon were retracted superiorly, and the intestine was retracted to the right. An attempt was made to grasp with fingers the base of the SMA. The peritoneum crossing the mesentery of the small bowel was incised, and the artery was dissected. At the same time, heparin (100 IU/kg weight) was infused through the peripheral intravenous catheter to all patients. Embolectomy was performed as the first choice for patients with open surgery, and for patients with failed embolectomy, it was followed by a bypass procedure with an autogenous vein graft.

Endovascular success was defined as the return of bowel perfusion without laparotomy or the return of bowel perfusion with laparotomy without the need for open revascularization by embolectomy or surgical bypass. Open revascularization was categorized as embolectomy or bypass graft. A failed embolectomy that was followed by a bypass procedure was recorded as a bypass graft.

Acute renal failure in the post-operative period was defined as a creatinine >1.5 mg/dl in patients with normal renal function or an increase of >20% in patients with chronic renal insufficiency. Pulmonary failure included patients who require intubation >72 h. Myocardial infarction included electrocardiogram-confirmed ST depression and elevation in the setting of hemodynamic compromise. The diagnosis of stroke was based on clinical examination in conjunction with cerebral imaging. Limited resection was defined by resection with a remaining length of small bowel >150 cm, which enables ingestion of food without causing the short-bowel syndrome. Mortality includes all in-hospital deaths.

Patient variables were compared using univariate statistics. Data are expressed as proportions for dichotomous variables and as the mean ± SD or median and interquartile range (IQR) (25–75th percentiles) for continuous variables. Differences between the two groups were determined by the *t-*test for parametric data and the Mann–Whitney *U* test for non-parametric data. The χ^2^ test was used for comparisons of nominal data, and Fisher's exact test was used when appropriate. Odds ratios were used to estimate the differences in the likelihood of death determined by operative, perioperative, and postoperative risk factors. Multivariate analysis was not used secondary to the overall low number of observed cases within each group. Statistical significance was set at *p* > 0.05. All analyses were performed using SPSS 24.0 software (SPSS Inc., Chicago, IL, USA).

## Results

During the 8-year study period, 41 patients with SMAE were treated with endovascular treatment of open surgery. The median patient age was 70 years (IQR, 60–77 years), and 56.1% were men. The patients' demographic data, clinical presentations, and characteristics are shown in Table 1. All patients had symptoms of acute abdominal pain, except 1 patient who presented with hematochezia. No patient had symptoms of chronic mesenteric ischemia. The median time between presentation and treatment was 48 h (IQR, 24–120 h), and 70.7% of patients were accompanied by atrial fibrillation.

**Table 1:** Demographic and clinical information of patients stratified by treatment type.

**Variables**	**All patients (*n* = 41)**	**Endovascular first (*n* = 14)**	**Open surgery first (*n* = 27)**	***p* ^a^**
Age, median (IQR), y	70 (60–77)	67 (60–80)	71 (58–76)	0.924
Male, %	56	57	56	0.923
Active smoking, %	24	7	33	0.142
Comorbidities, %				
Hypertension	66	86	56	0.113
Diabetes mellitus	44	43	44	1.000
Atrial fibrillation	71	79	67	0.665
Coronary artery disease	44	64	33	0.058
Congestive heart failure	12	14	11	1.000
Chronic renal insufficiency	12	14	11	1.000
ASA>3	29	7	41	0.060
History of embolic event	5	0	7	0.539
Duration of symptoms onset to treatment, median (IQR), h	48 (24–120)	48 (12–120)	48 (24–96)	0.674
Abdominal pain, %	98	100	96	1.000
Emesis, %	71	79	67	0.665
Diarrhea, %	24	21	26	1.000
Hematochezia, %	22	7	30	0.211
WBC count, mean ± SD, × 10^3^/dL	15 ± 9	12 ± 6	17 ± 11	0.07
Neutrophil ratio median (IQR), %	87 (82–90)	87 (71–89)	87 (82–91)	0.413
Urea, mean ± SD, mmol/L	9 ± 5	8 ± 6	9 ± 4	0.574
Creatinine, mean ± SD, umol/L	110 ± 140	135 ± 214	92 ± 41	0.386
Potassium, mean ± SD, mg/dL	3.9 ± 0.6	3.8 ± 0.6	4.0 ± 0.6	0.192
Alanine transaminase, median (IQR), U/L	17 (11–25)	14.5 (9.8–22.0)	20.5 (12.3–37.8)	0.138
Aspartate transaminase, median (IQR), U/L	23 (16–46)	18.0 (13.6–35.1)	31.8 (18–53.2)	0.084
Lactate, median (IQR), mmol/L	3.4 ± 1.9	2.7 ± 2.8	3.6 ± 1.6	0.408
D-dimer (IQR), mg/L	2.2 (1.2, 3.2)	2.2 (1.5, 5.7)	2.15 (1.1, 3.0)	0.877
Ph	7.41 ± 0.03	7.42 ± 0.05	7.41 ± 0.04	0.597

*IQR, interquartile range; SD, standard deviation; ASA, American Society of Anesthesiologists; WBC, white blood cell*.

a *Denotes comparisons between endovascular therapy and open surgery*.

Endovascular therapy was initiated in 14 patients (34.1%).

Only minor differences were found when the groups treated with endovascular therapy and open surgery were compared. Patients treated with endovascular therapy may have more comorbidities, such as coronary artery disease, but without a difference in value (64% vs. 33%; *p* = 0.058). In addition, the white blood cell (WBC) count of the endovascular first was not different from that of the traditional cohort (12 ± 6 vs. 17 ± 11; *p* = 0.07). A comparison of the symptoms and other clinical characteristics during admission found no difference in values. The duration of symptoms before treatment was longer in the patients treated with open surgery first, but without a difference in value (median, 48 vs. 24 h; *p* > 0.05).

Descriptive variables for operative management are listed in Table 2. The duration of the initial endovascular procedure was much shorter than that of traditional therapy (102 vs. 210 min; *p* < 0.05), as was the blood loss of these two groups (20 vs. 200 ml; *p* < 0.05). The technical success of the endovascular first group was 93%, with 1 patient not having bowel perfusion because the guiding wire could not pass through the lesion, and the patient finally underwent surgical bypass. For the rest of the patients, the primary mode of endovascular therapy was mechanical thrombectomy, comprising 92% of the population. Of those patients, 50% of the patients were treated with mechanical thrombectomy without thrombolysis, 3 patients were treated with mechanical thrombectomy adjunctive percutaneous transluminal angioplasty (PTA) and stenting, and 3 patients had adjunctive catheter-directed thrombolysis after mechanical thrombectomy. One patient (8%) was treated with primary PTA and stenting. Twenty-seven patients underwent open surgery first, and bowel resection alone without revascularization was used in 9 patients (33%). In these 9 patients, 5 had a limited segment of bowel gangrene, and SMA embolization was restricted to side branches. Four patients had extensive bowel necrosis. Embolectomy or surgical bypass was considered to have no effect on improving intestinal blood supply, so they had an extensive bowel resection, and 3 of them had enterostomy without primary enteric anastomosis. A total of 18 (67%) patients underwent embolectomy, including 9 who required segmental resection.

**Table 2:** Comparison of operative management stratified by treatment type.

**Variables**	**Endovascular first (*n =* 14)**	**Open surgery first (*n =* 27)**	***p* ^a^**
Procedure duration, median (IQR), min	102 (73–179)	210 (170–246)	0.000
Blood loss, median (IQR), ml	20 (20–42.5)	200 (100–300)	0.000
**Endovascular first treatment, %**			
Mechanical thrombectomy alone	46		
Adjunctive catheter-directed thrombolysis	23		
Adjunctive PTA/stent	23		
Primary PTA/stent	8		
Endovascular technical success, %	93		
Open surgery first treatment, %			
Embolectomy	67		
Bypass	0		
Bowel resection only	33		
**Laparotomy**			
Ischemic bowel requiring resection, %	36	67	0.06
Bowel resection, median, mean ± SD, cm	1.4 ± 0.9	2.1 ± 1.2	0.194

a *Denotes comparisons between endovascular therapy and open surgery*.

Of patients who underwent endovascular therapy first, 64% avoided bowel resection, had no difference compared to the open surgery first group (32%, *p* = 0.06), and of those requiring bowel resection, a median of 1.4 m of bowel was resected compared with 2.1 m for open surgery first group (*p* > 0.05).

Post-operative complications are summarized in Table 3.

**Table 3:** Outcomes of treatment stratified by treatment type.

**Variables**	**Endovascular first (*n =* 14)**	**Open surgery first (*n =* 27)**	***p* ^a^**
**Complications**			
Acute kidney injury, %	21	52	0.061
Pulmonary failure	0	19	0.224
Myocardial infarction, %	21	30	0.849
Stroke, %	7	7	1.000
Second-look operation	36	4	0.02
Sepsis	21	41	0.374
Limited resection	79	63	0.506
Bowel rest time	6 (5–10)	11 (8–17)	0.022
Hospital stay median (IQR), day	11 (9–20)	19 (11–28)	0.159
ICU time median (IQR), day	1 (0–4)	5 (2–14)	0.004
Ventilator used time median (IQR), hour	0 (0–9)	11 (1–50)	0.011
Mortality, %	21	33	0.665

a *Denotes comparisons between endovascular therapy and open surgery*.

One (4%) patient underwent a second-look operation in the open surgery group because of wound infection; however, reoperation was needed for 36% of patients in the endovascular first surgery group because of bowel gangrene (*p* < 0.05). The endovascular group had less time needed for bowel rest, stayed in the intensive care unit (ICU), and ventilator use. The in-hospital mortality rate showed no difference between the two groups (21% vs. 33%, *p* > 0.05). Post-operative complications are summarized in Table 3.

Table 4 lists univariate factors associated with death in patients with AME. A history of chronic renal insufficiency, ASA >3, initial WBC >12 × 10^3^/dl, creatinine >92 μmol/dl, and urea >6.2 mmol/L were associated with an increased risk of death. During the perioperative period, the length of necrotic bowel requiring resection >2 m also positively affected overall mortality. In the postoperative period, acute renal failure, pulmonary failure, myocardial infarction, and stroke were significantly associated with death.

**Table 4:** Univariate associations with mortality among superior mesenteric artery embolism patients.

**Variable**	**OR (95% CI)**	***P***
Age		
>70	2.83 (0.69–11.60)	0.141
≤ 70 (ref)	1.0	–
Gender		
Male	0.71 (0.18–2.73)	0.61
Female (ref)	1.0	–
Chronic renal insufficiency	14.00 (1.37–143.59)	0.03
Coronary artery disease	1.42 (0.37–5.47)	0.61
Atrial fibrillation	1.35 (0.29–6.21)	0.99
Duration of symptoms > 24 h	0.71 (0.18–2.73)	0.61
ASA > 3	12.50 (2.53–61.81)	0.003
WBC >12* 10^3^/dL	9.69 (1.07–87.44)	0.05
Creatinine >92 umol/dL	11.08 (1.80–68.40)	0.01
Urea >6.2 mmol/L	1.75 (1.21–2.54)	0.02
potassium >4 mmol/L	3.75 (0.72–19.64)	0.231
D-dimer>2.6 mg/L	3.96 (0.80–19.67)	0.19
**Type of therapy**		
Traditional	1.83 (0.40–8.27)	0.67
Endovascular first (ref)	1.0	–
Necrotic bowel	1.14 (0.29–4.44)	0.85
Length of necrotic bowel requiring resection >2 meters	8.67 (1.67–44.94)	0.02
Acute renal failure	7.88 (1.69–36.72)	0.01
Pulmonary failure	14 (1.37–143.59)	0.03
Myocardial infarction	8.75 (1.84–41.60)	0.01
Stroke	21.74 (1.03–459.93)	0.02

*CI, Confidence interval; OR, odds ratio; ASA, American Society of Anesthesiologists; WBC, white blood cell*.

Overall, endovascular treatment had no survival benefit. Comparison in patients who had bowel necrosis showed no significant difference in mortality for endovascular therapy first (40%) vs. traditional therapy (28%; *p* = 0.60); however, the endovascular first group had lower mortality than the open surgery group for patients without bowel necrosis, but the difference was not significant (11% vs. 44%; *p* = 0.14).

## Discussion

Acute mesenteric ischemia remains an acute surgical emergency with an overall poor prognosis. SMAE is the most common cause of AMI (5). This is closely related to the anatomical structure of the SMA, which originates from the anterior wall of the abdominal aorta. It emits at a small angle, approximately parallel to the aorta, the blood flow direction is the same, and the diameter is relatively large, making it easy for emboli to enter (6). However, its clinical outcome is reported to be better than mesenteric arterial thrombosis and non-occlusive mesenteric ischemia (7, 9). However, mortality rates remain high at 30–54%, excluding patients who received supportive care, according to other studies (8–10). Many etiological factors, such as valvular heart disease, cardiac arrhythmia, left atrial thrombosis, aortic calcification, previous stenotic lesions, iatrogenic causes, and tumors, can cause SMAE, and most emboli originating from the heart are secondary to atrial fibrillation (2). The result was similar in this study, with 71% of patients having atrial fibrillation. This may be because of inadequate anticoagulation, and only 21% of atrial fibrillation patients in this study had anticoagulation treatment before this administration. Although the gold standard of diagnosis remains arteriography historically, CT angiography (CTA) was used in essentially all patients because it is quick, non-invasive, and more readily differentiates SMAE from other abdominal diseases. Additionally, CTA provides additional information, such as bowel wall thickening, bowel wall enhancement, presence of free air, pneumatosis intestinalis, and peritoneal fluid collection. This examination can evaluate the origin of the SMA and access vessels before arteriography are performed (9, 11).

Several clinical factors have been suggested as prognostic factors by previous studies. The first is the duration of symptom onset to diagnosis and revascularization. Several studies reported that the patients' outcomes significantly improved with the diagnosis within 24 h (12, 13). There were 79 patients with SMAE in these two studies, with significant improvement in mortality from 88–90% to 32–57% when the diagnosis was made within 24 h. However, in another study, there was no association between symptom duration and mortality (9). In our cohort, there was no improvement in mortality even when the patients received treatment within 24 h. Obviously, the extent of intestinal ischemia progression is much more important than symptom duration. The extent of ischemia does not always correlate with time because it depends on the level of occlusion, patient collateral circulation, and splanchnic autoregulation (9). In most cases of acute SMAE, emboli lodge ~6–8 cm beyond the SMA origin, distal to the origin of the middle colic artery. Nevertheless, atheroembolic emboli are more likely to be smaller, so they could lodge in the more distal mesenteric circulation, which perhaps has a better prognosis (14). In this study, all 5 patients who had distal embolism survived, even though they had limited segments of bowel gangrene. Patients with SMAE often have a variety of medical comorbidities, and their age and comorbidities may affect the prognosis. Other studies (11, 15) reported that increasing age, history of peripheral arterial disease, coronary artery disease, initial lactate >2.2 mmol/L, and maximum lactate level during the perioperative period and bowel necrosis were associated with an increased risk of death and bowel loop dilation on CT scans was a predictive factor for irreversible transmural intestinal necrosis. In this study, comorbidities, WBC count, and renal function of the patients were associated with an increased risk of death, but increasing age, initial lactate level, and necrotic bowel showed no statistically significant difference. Although the presence of bowel necrosis was not a prognostic factor, the length of necrotic bowel requiring resection >2 m also positively affected overall mortality. In short, we think the most important risk factors are the extent and severity of ischemic bowel.

To address poor outcomes of SMAE, a multimodal approach focusing on (1) the removal of non-viable segments of ischemic bowel (2) the preservation of the non-necrotic intestine with revascularization, and (3) medical treatment to prevent progression to multiorgan failure (1, 10). Consistent with our study, the importance of revascularization is highlighted. Generally, surgical treatment involves exploratory laparotomy and assessment of the viability of the bowel (2). While surgery is necessary for advanced patients with non-viable bowel, endovascular treatment has several advantages, such as the avoidance of general anesthesia and the use of laparotomy in high-risk patients (16). Additionally, conservative treatment, such as bowel rest, parenteral nutritional support, and nasogastric drainage, may be adopted in selected patients with SMAE, but most importantly, close monitoring for bowel gangrene and adequate anticoagulation is necessary (9).

The gold standard of open surgery is an open transperitoneal approach *via* a full midline incision of the abdomen. After the confirmation of the location of the lesion, surgical embolectomy or bypass graft and bowel assessment for viability were performed. The use of prosthetic conduits or patches is contraindicated with intestinal perforation and obvious intestinal contamination. The saphenous vein or femoral vein can be utilized in this setting (17). Embolectomy was the first choice for patients with SMAE who underwent open surgery in other studies (2, 18), as in our center, because embolectomy spends a shorter time for revascularization than bypass surgery. In our cohort, embolectomy was performed in all patients who needed revascularization, and bypass by the saphenous vein was used in only 1 patient who had an endovascular failure.

One of the most important goals of open surgery is to evaluate the viability of the small bowel. Clinical judgment, fluorescein injection, and Doppler ultrasonography are useful techniques to evaluate intestinal viability (2). Bulkley et al. (19) reported that the accuracy of clinical judgment was 89%. In our study, we observed viability by clinical judgment, with no patient who underwent reintervention for bowel necrosis in the open surgery group. This may indicate that clinical judgment is still an accurate and convenient method. In addition, laparoscopic exploration may be considered to evaluate intestinal viability.

For patients with no clear evidence of bowel necrosis, endovascular treatment can be a promising alternative and minimally invasive interventional approach (17). Some scholars have attempted to use thrombectomy devices for SMAE and have achieved good results; however, these retrospective series were small (17, 20). Thrombectomy devices, such as the Rotarex debulking Device (Straub Medical, Wangs, Switzerland), AngioJet thrombectomy catheter (Solent Omni; Boston Scientific, Marlborough, MA, USA), and Solitaire FR revascularization device (Covidien, Irvine, CA, USA), are used for such treatments (16, 21, 22). In our cohort, mechanical thrombectomy performed with the Rotarex system was the first choice. After debulking, balloon dilatation, thrombolysis, and stent implantation were considered according to angiography results. The Rotarex system has been widely used in thrombotic diseases of lower limb arteries and sometimes in renal arteries (23, 24). This rotational thrombectomy is capable of precluding and replacing thrombolysis and may be an effective and safe modality for restoring blood supply to the bowel fast (24). There are reports supporting that endovascular therapy affords lower laparotomy rates, significantly smaller bowel resection at the time of surgical exploration, and lower rates of renal and pulmonary failure (4, 5). On the other hand, endovascular preference treatment may raise several concerns that could potentially worsen patient outcomes. First, endovascular treatment cannot evaluate the viability of the small bowel directly, and the exploration was reserved only for peritoneal findings on abdominal examination or clinical deterioration, which may delay the resection of necrotic bowels and increase sepsis. Second, endovascular failures could delay revascularization and worsen outcomes (5, 11). In this series, although the endovascular first group had advantages in duration surgery time, blood loss, bowel rest time, ICU stay time, and ventilator use, there was no difference in the in-hospital mortality rate, bowel resection rate, or length between these two groups. This may be because of the high reoperation rate for the endovascular first group with 5 patients (36%), except that 1 patient who underwent reoperation because of endovascular failure, and the remaining patients returned bowel perfusion but still underwent reoperation for necrosis bowel resection. Of these 4 patients, 50% died because of sepsis, and 1 patient had extensive bowel necrosis and finally underwent enterostomy. On the other hand, the mortality of patients who underwent bowel resection and revascularization in the open surgery group was 28%, which seems better than the 4 patients mentioned above without significance. For the patients who had no bowel necrosis and underwent endovascular treatment, the mortality was much lower (11%). Therefore, we think that the 4 patients who had bowel resection even after successful revascularization may have had bowel necrosis before endovascular treatment. These patients had no evidence of bowel necrosis we mentioned above, but when we retrospectively analyzed their neutrophil count, it was much higher in these patients than in other patients who underwent endovascular treatment (13 ± 7 vs. 7 ± 3, *p* < 0.05). In AMI, leukocytosis is often present because partial- or full-thickness bowel necrosis allows bacterial translocation and subsequent leukocytosis (17). In our study, an initial WBC count >15 × 10^3^/dl and neutrophil count >13 × 10^3^/dl were associated with an increased risk of bowel necrosis, with ORs (95% CI) of 17.50 (3.02–101.54) and 4.8 (1.20–19.13), respectively. Therefore, the increase in WBC count or neutrophil count may indicate the presence of bowel necrosis at first. For these patients, endovascular treatment alone should be carefully selected. Of course, this also needs to be judged with other signs of bowel necrosis.

In addition, the safety of endovascular treatment was concerned, mainly about hemorrhagic stroke, gastrointestinal bleeding, and access-related bleeding. In fact, bleeding complications were relatively low according to other reports (4, 11, 20). Arthurs et al. (11) reported complications of access-related bleeding for 9%. Access-related complications may be associated with the access site and the diameter of the introducer used. Generally, we choose femoral access first, and brachial access is used after a failed femoral approach. In this cohort, 57% of patients completed the treatment successfully through femoral access, and the rest of the patients used brachial access with a 6F introducer. No deleterious effects of endovascular therapy were identified in any patients. Additionally, careful attention should be given when performing mechanical thrombectomy, with the potential risk of SMA pseudoaneurysm formation (25).

Limitations of this study deserve mention. This study is inherently subject to selection bias, as we chose healthier and mild patients to undergo endovascular repair while deteriorating, unwell patients with bowel necrosis may have proceeded to laparotomy directly. Although selection bias was evident, there were few measurable differences between the two groups of patients. Second, we are limited by the relatively small number of patients enrolled, and there may be type II errors. Another limitation is that the long-term survival of patients and quality of life based on treatment were not investigated. Despite these limitations, several strengths exist. This represents the largest single-center experience using endovascular therapy for the treatment of SMAE.

## Conclusions

Superior mesenteric artery embolism remains one of the most lethal diseases treated by vascular surgeons. Chronic renal insufficiency, ASA >3, and indicators suggesting severe bowel necrosis (WBC >12 × 10^3^/dl, necrotic bowel >2 m) positively affected overall mortality. Endovascular therapy has altered the management of SMAE, and it may be adopted in selected patients who are not at risk for bowel necrosis. However, avoidance of bowel necrosis patients and close monitoring for bowel necrosis is important.

## Data Availability Statement

The raw data supporting the conclusions of this article will be made available by the authors, without undue reservation.

## Ethics Statement

The studies involving human participants were reviewed and approved by Institutional Ethical Review Board of Beijing Friendship Hospital. The patients/participants provided their written informed consent to participate in this study.

## Author Contributions

WL and HF contributed to the conception and study design and obtained funding. WL and SC contributed to writing the article. ZZ, RZ, XC, and BL contributed to the critical revision of the article. WL helped in the data collection, analysis, and interpretation. All authors contributed to the article and approved the submitted version.

## Conflict of Interest

The authors declare that the research was conducted in the absence of any commercial or financial relationships that could be construed as a potential conflict of interest.

## Publisher's Note

All claims expressed in this article are solely those of the authors and do not necessarily represent those of their affiliated organizations, or those of the publisher, the editors and the reviewers. Any product that may be evaluated in this article, or claim that may be made by its manufacturer, is not guaranteed or endorsed by the publisher.
